# Transcriptomic and metabolomic insights from functionalized V-shaped *Lactiplantibacillus plantarum* towards mitigating *Candida albicans* virulence

**DOI:** 10.1016/j.bioflm.2025.100321

**Published:** 2025-10-01

**Authors:** Satish Kumar Rajasekharan, Athira Venugopal, Hamitha Chinganadi Hameed, Jennessa Jacob, Vinothkannan Ravichandran, Doron Steinberg, Adi Faigenboim, Chaitany Jayprakash Raorane, Moshe Shemesh

**Affiliations:** a Department of Biotechnology, School of Bioengineering, SRM Institute of Science and Technology, Kattankulathur, 603203, India; b Department of Food Science, Institute of Postharvest Technology and Food Sciences, Agricultural Research Organization (ARO), Volcani Institute, Rishon LeZion, 7528809, Israel; c Biofilm Research Laboratory, Institute of Biomedical and Oral Research (IBOR), Faculty of Dental Medicine, The Hebrew University of Jerusalem, Jerusalem, 9112102, Israel; d Center for Drug Discovery and Development (CD3), Amity University Maharashtra, Mumbai - Pune Expressway, Bhatan, Panvel, Mumbai, Maharashtra, 410206, India; e Institute of Plant Sciences, The Volcani Institute, Rishon LeZion, 7528809, Israel; f School of Chemical Engineering, Yeungnam University, Gyeongsan, 38541, Republic of Korea

**Keywords:** Probiotic biofilm, Geometrical structuring, Lactobacilli, Hyphae, Microcolony

## Abstract

The genomic capacity of probiotic Lactobacilli enables producing bioactive metabolites that represent promising alternatives to antibiotics for combating food-borne and multi drug-resistant pathogens. The current study characterizes a global antagonistic potential of the probiotic bacterium *Lactiplantibacillus plantarum*, which demonstrates unique multicellular structuring triggered in response to acidic environments. We demonstrate the inhibitory potential of postbiotics derived from the V-shaped structured *L. plantarum* (VSLP) against *Candida albicans*. Using the liquid chromatography-mass spectrometry (LC-MS) profiling, we further identify different antibiofilm metabolites secreted during the VSLP formation. Transcriptomic studies reveal a notable upregulation of genes associated with the biosynthesis of aromatic amino acids such as *serD, ywqE2, trpB, trpA*. Furthermore, significant differential expressions were observed in genes within the biosynthetic gene cluster (BGCs) identified through antiSMASH analysis of *L. plantarum* genome. In addition, two novel VSLP metabolites (bluensomycin and majoroside F6) functioned as inhibitors of Ras-adenylate cyclase pathway that control biofilms and hyphae in yeast. We suggest that functionalized probiotic cells, such as VSLP, may effectively control the pathogenic microorganisms that provide a staple basis for developing future therapeutic probiotics.

## 1 Introduction

Functional probiotics may provide health benefit to the host when administered in sufficient quantities [1]. Among most prominent probiotic microorganisms are Gram-positive lactic acid bacteria (LAB), which mainly belong to the *Lactobacillus* and *Bifidobacterium* genera [2]. The essential probiotic requirement in terms of the health benefits is a positive influence on digestion and immune systems [3]. Moreover, probiotics also have a protective role, directly competing with pathogens through releasing antimicrobial substances like organic acids, bacteriocins and hydrogen peroxide [4]. Nevertheless, it appears that probiotic cells must be often functionalized to exert utmost beneficial effects within the host organism.

The probiotic Lactobacilli and their beneficial metabolites are increasingly proposed as an effective therapy to combat pathogenic microorganisms with increased tolerance to antibiotic therapy [[5], [6], [7]]. Occasionally, such probiotic therapy appears to be very effective against different bacterial and fungal pathogens [7]. Studies have shown that live Lactobacilli exhibit significant antagonistic activity against the pathogenic cells in coculture models [8,9]*.* Other studies postulated the antimicrobial effects of the *Lactobacillus* cell-free supernatant against biofilms formed by pathogens such as *Escherichia coli*, *Staphylococcus aureus*, and *Candida albicans*. [10,11]. It has also been demonstrated that the physiological state of Lactobacilli may govern eliminating pathogens by producing postbiotic substances with potent antimicrobial activities [11,12].

In previous study, we observed that *Lactiplantibacillus plantarum* undergoes unusual V-shaped multicellular structuring (VMS) in response to acidic pH stress. VMS is characterized by angular chains of several undivided cells locked in a V-conformation. This morphology likely represents an adaptive strategy, where stalled cell division and geometrical bundling provide potential bio-shielding against acidic stress and contribute to a ‘bottle effect,’ enabling cells to retain their V-shape while forming robust biofilms and thereby increasing resistance to environmental stresses [12]. Mechanistically, we showed that VMS enhances the survivability of *L. plantarum* under acidic conditions and promotes probiotic or antagonistic biofilm formation through the LuxS-dependent quorum-sensing pathway associated with autoinducer-2 (AI-2) production [13]. Such structured multicellularity strengthens the antagonistic activity against pathogenic yeasts like *Candida albicans*, which coexist with Lactobacilli in acidic niches such as the vaginal microbiota [12,13]. Thus, the current study elucidates the mode of antimicrobial activity of the fermentation products (postbiotics) obtained from V-shaped structured *L. plantarum* (VSLP) cells, tested against a pathogenic yeast model. We subsequently identify new antagonistic molecules derived from the VSLP cells that could potentially be used for future drug designing.

## 2 Materials and methods

**Microbial strains and growth conditions:** *Candida albicans* strain (ATCC 90028/MTCC 3017) and *Lactiplantibacillus plantarum* strain (MTCC 1407) were obtained from the Microbial Type Culture Collection (MTCC), India. *L. plantarum* was maintained in de Man, Rogosa, and Sharpe (MRS) broth at 37 °C with shaking at 150 rpm, or on MRS agar (HiMedia, India) incubated at 37 °C. *C. albicans* was maintained in potato dextrose broth (PDB) under the same growth conditions (37 °C, 150 rpm shaking), or on potato dextrose agar (PDA) (HiMedia, India) incubated at 37 °C.

**Fig. 1:**
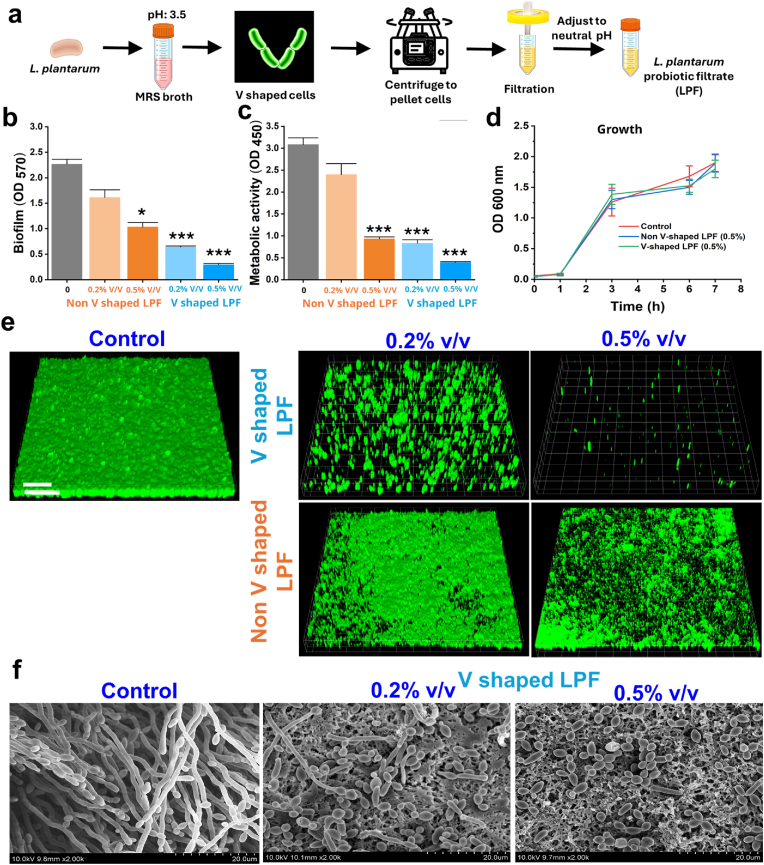
Effect of LPF on biofilm formation by bacterial and yeast pathogen. (a) Schematic representation of Lactobacilli probiotic filtrate (LPF) preparation from *L. plantarum* cultures. (b) Biofilm formation by *C. albicans* with or without non-V shaped and V -shaped LPF treatment (0.25–0.5 % v/v), quantified by crystal violet assay. ∗∗∗p < 0.001 vs. non-treated controls. (c) Metabolic activity of *C. albicans* biofilms assessed by XTT reduction assay in the presence or absence of LPF. ∗∗∗p < 0.001 vs. controls. (d) Growth profile of *C. albicans* biofilms with or without non-V shaped and V -shaped treatment (OD600). (e) Confocal laser scanning microscopy (CLSM) images of *C. albicans* biofilms stained with carboxyfluorescein diacetate succinimidyl ester; excitation 488 nm, emission 500–550 nm; scale bar: 100 μm. (f) Scanning electron microscopy (SEM) images of *C. albicans* hyphae grown on nylon membranes, treated with or without V -shaped LPF. Samples were fixed in 2 % glutaraldehyde and 2 % formaldehyde, dehydrated in graded ethanol, and imaged using a Hitachi S-4100 SEM at 15 kV. Scale bar: 10 μm. (For interpretation of the references to color in this figure legend, the reader is referred to the Web version of this article.)

**Preparation of Lactobacilli probiotic filtrate:** *L. plantarum* grown in MRS set at pH 3.5 were pelleted to remove the cells and the filtrate was filter sterilized using a 0.2 μm filter (Sigma Aldrich, USA) and pH was adjusted to neutral. The resulting filtrate was designated as V-shaped Lactobacilli probiotic filtrate (V-shaped LPF) (Fig. 1a)

**Biofilm inhibition assay:** Biofilm quantification assays were conducted in 96-well polystyrene plates in accordance with previous descriptions [11,12,14]. In brief, *C. albicans* cells were grown in PDB overnight at 150 rpm at 37 °C then inoculated in fresh PDB. After adjusting to OD600 = 0.01, the cultures were again seeded on 96-well polystyrene plates with or without V-shaped LPF (0.25–0.5 % v/v) and incubated for 48 h at 37 °C. The surface attached biofilms were quantified at OD 575 nm by the standard crystal violet quantification method, and the growth was evaluated at 600 nm after 48 h.

***Caenorhabditis elegans* Toxicity Assay:** *Caenorhabditis elegans* wild-type strains were cultured and maintained on the nematode growth medium (NGM) with *Escherichia coli* OP50 as a feed. In order to assess the toxicity of V-shaped LPF (0.5–2 % v/v), synchronized adult nematodes were cultured and examined in a 96-well microliter plate as previously described [15]. The control and treatment groups (each consisting of approximately 30 nematodes per well) were suspended in liquid M9 buffer and observed for 7 days. The live or dead nematodes were counted using the Nikon fluorescent microscope (Nikon Eclipse Ti2, Japan) using DIC and DAPi (blue LED light) filters, and the survival percentage was determined.

**Ibidi microcolony assay:** Single cell microcolony assay was conducted in μ-Slide I Luer (Surface Modification: ibidi Treat, channel Height: 0.4 mm) (Ibidi, Germany). Briefly, *C. albicans* cells (100 cells) in RPMI -1640 were seeded on the ibidi channel and allowed to grow as a single cell microcolony. For treatment, VSLP cell chains (approximately 1000 cells) were suspended in RPMI 1640 and added to the wells of the ibidi slide following which the slides were incubated at 37 °C for 24 h [16]. After incubation, the cells were imaged and recorded using a light microscope.

**XTT assay:** The XTT [2,3 -bis (2 -methoxy - 4 -nitro - 5 -sulfophenyl) -2H -tetrazolium - 5 - carboxanilide sodium salt] reduction test was used to measure the metabolic activity of *C. albicans* biofilm cells. Overnight cultures of *C. albicans* in PDB were re-inoculated into fresh PDB with or without V-shaped LPF and incubated at 37 °C for 24 h. Following incubation, XTT-menadione solution was added to the plates, which were then kept in the dark at 37 °C for 2 h to allow the development of orange color [17,18]. The supernatant was subsequently measured at 450 nm.

**Scanning electron microscopy:** Images of the biofilm cells attached to the nylon membrane were taken using a scanning electron microscope (SEM). Nylon membranes were cut into 0.5 × 0.5 cm pieces and placed in 96-well plates containing *C. albicans* grown with or without V-shaped LPF. Fixation of nylon membrane-attached cells was performed using glutaraldehyde (2 %) and formaldehyde (2 %) for 24 h, followed by post-fixation using sodium phosphate buffer, osmium, an ethanol series (50 %, 70 %, 80, 90, 95, and 100 %) and isoamyl acetate. Using a S-4100 scanning electron microscope (Hitachi, Japan) at a voltage of 15 kV, cells were examined and imaged after critical-point drying.

**Confocal microscopy:** Biofilms were stained with carboxyfluorescein diacetate succinimidyl ester (Invitrogen, Molecular Probes, Inc., Eugene, OR, USA). Planktonic cells were removed by washing with PBS three times, and static biofilms were visualized by excitation using an Ar laser 488 nm (emission wavelengths 500–550 nm) under a confocal laser microscope (Nikon Eclipse Ti, Tokyo) using a 20× objective. Color confocal images were constructed using NIS-Elements C version 3.2 (Nikon Eclipse). For each experiment, at least 20 random positions in three independent cultures were chosen for microscopic analysis [14]**.**

**LC-MS (liquid chromatography-mass spectrometry:** Culturing and metabolite extraction were done for LPS extracted from *L. plantarum* grown at pH 3.5. Sample preparation was done by concentrating and purifying the metabolites to increase the analyte concentration. Sample injection was done with optimized parameters. The chemical constituents of the tested extracts were identified using an HR-LCMS-QTOF (Agilent Technologies, USA), employing mass spectrometry for the detection and characterization of metabolites. LC-MS data was analyzed for peak identification and spectral matching. Then, spectra were compared with databases to identify metabolites.

**RNA isolation:** For global gene expression analysis, an overnight culture of *L. plantarum* (OD600 around 2) was reinoculated in MRS media with adjusted pH to either 6.5 or 3.5, and the cells were harvested after 4 h of growth. Total RNA was extracted by the Tri-Reagent (Sigma-Aldrich, St. Louis, MO, USA) protocol wherein the bacterial cultures were treated with 2 mg/mL of lysozyme in PBS for 10 min at 37 °C, 1 mL RNA protect (QIAGEN, Hilden, Germany) for 5 min, and centrifuged at 5000×*g* at 4 °C for 10 min. The pellets were resuspended in 1 mL of TriReagent (Sigma-Aldrich, St. Louis, MO, USA) and were disrupted using acid-washed glass beads using three 45-s disruption cycles at 4500 rpm using a FastPrep cell disruptor (BIO101, Savant Instruments) with a pause of 5 min on ice after each cycle. The supernatants collected after centrifugation for 2 min at 14,000×*g* were transferred to new 1.5 mL Eppendorf tubes and 200 μL chloroform was added, followed by thorough vertexing for 15 s to ensure complete mixing. After 15 min at room temperature, the samples were centrifuged at 21,000×*g* for 15 min 300 μL of the RNA-containing upper phase was transferred to a fresh 1.5 mL Eppendorf tube to which an equal volume of isopropanol was added. The tubes were thoroughly mixed by inverting them and then left at room temperature for 30 min before centrifugation at 4 °C at 21,000×*g*. The RNA pellets were washed twice with 1 mL of 75 % ethanol, dried, and then dissolved in 50 μL of DNase- and RNase-free ultrapure water (Bio-Lab Ltd, Jerusalem, Israel). RNA concentration was determined by nanodrop prior to sending for sequencing.

**Global transcriptomic analysis:** Libraries for Illumina sequencing were made with the NEBNext rRNA Depletion Kit (bacteria) prep kit (New England Biolabs, USA) according to the manufacturer's instructions. RNA sequencing was performed on the Illumina NextSeq 2000 platform using single‐end 120 bp sequencing. Cutadapt (v3.5), was used to trim low quality and technical bases, using a quality threshold of 32 for both ends and removing poly-G sequences and adapter sequences were then removed from the 3′ end. Reads were further filtered by overall quality, using fastq_quality_filter (v0.0.14, FASTX package), with a quality threshold of 20 at 90 percent or more of the read positions.

Processed reads were aligned to the reference genome retrieved from the NCBI database (GCF_009913655.1_ASM991365v1) using Bowtie2 (v2.2.5) [19]. Counting was then performed for alignments with a mapping quality at least 10, at gene level, with with htseq-count (v0.6.0) [20], using the reverse direction.

Normalization and differential expression analysis were done with the DESeq2 package (v1.30.0) [21] after removal of genes with less than 10 counts over all samples were filtered out, then size factors and dispersion were calculated. Normalized counts were used for several quality control assays, such as counts distributions and principal component analysis, which were calculated and visualized in R (R version 4.0.4, with packages RColorBrewer_1.1–2, pheatmap_1.0.12, ggplot2_3.3.3 and ggrepel_0.9.0). Comparing Acidic (*L. plantarum* in MRS pH 3.5) to Control was tested with default parameters, taking the significance threshold as padj<0.05.

We used KOBAS 3.0 (http://bioinfo.org/kobas/) to find statistically significant enrichment of differentially expressed genes in the KEGG pathway database and Gene Ontology (GO) categories (Bu et al., 2021)

**qPCR analysis**: To analyse the gene expression of *C. albicans* treated with V-shaped LPF, cultures were grown in RPMI 1640 medium in the presence of V-shaped LPF. After 6 h of incubation, total RNA was isolated using the TRIzol method. Isolated RNA was quantified and cDNA was synthesized using a commercial cDNA synthesis kit (Abclonal) on a thermocycler (Dicetouch TP350, Takara, Japan). The synthesized cDNA was then used as a template for quantitative PCR with SYBR Green dye (Genious 2X SYBR Green Fast qPCR Mix). The reaction was performed in a real-time PCR system (Applied Biosystems, USA) with the following thermal cycling conditions: initial denaturation at 95 °C, followed by 40 cycles of 95 °C for 5s. For analysis ACT1 was used as the housekeeping gene by Ct method.

**Molecular docking analysis**: Molecular docking study was executed by Schrodinger version 9.3. The ligands were flexible to rotate within the binding poses, while receptor was kept rigid. A grid was generated with close proximity to active sites of adenylate cyclase and docking was performed by Glide (XP model). The grid maps representing the center of active site pocket for the ligand were calculated with Autogrid. Glide module of Schrodinger 9.3 was used for docking Mannich bases with the crystal structure of adenylate cyclase (Code 1FX2) retrieved from the Protein Data Bank (PDB) (https://doi.org/10.2210/pdb1fx2/pdb) as described [22,23]. Finally, the results generated were visualized by PyMOL viewer for analysis of minimum binding energy (Kcal/mol), Ki (Inhibition constant) value (μM), and hydrogen and hydrophobic interaction of the docked inhibitor to the modeled structure.

**Statistical analysis:** The student's *t*-test was used to determine the significance of differences between treated and non-treated samples. Statistical significance was accepted for *p* values < 0.05, and significant changes are indicated using asterisks in figures (∗ = *p* < 0.05; ∗∗ = *p* < 0.01; ∗∗∗ = *p* < 0.001).

## 3 Results and discussion

An emerging strategy for developing functional or medicinal foods is the use of probiotics or their fermentation products (postbiotics) to inhibit harmful microorganisms [24,25]. *L. plantarum*, a prospective biofilm-forming probiotic bacterium, has an enormous capacity to control pathogens [26]. Biofilm formation by *L. plantarum* is governed by numerous environmental factors [27]. We have recently shown that *L. plantarum* forms a unique V-shaped multicellular structuring (VMS) with superior biofilm forming ability accompanied with robust antagonistic activity against pathogenic yeast [12]. We termed the subpopulation of cells contributing to VMS as V-shaped structured *Lactiplantibacillus plantarum* (VSLP) cells.

To characterize the antagonistic potential of the VSLP cells, we generated postbiotic filtrates from the cells and labelled them as V-shaped Lactobacilli probiotic filtrate (V-shaped LPF) (Fig. 1a). Next, we assessed the antibiofilm activity of Non V shaped (pH 6.5) and V shaped (pH 3.5) Lactobacilli probiotic filtrate (V shaped LPF) on pathogenic *C. albicans.* We noted that V-shaped LPF could significantly inhibit the biofilm formation by the yeast cells (Fig. 1b).

Metabolic analysis further supported the biofilm inhibitory profile (Fig. 1c). We observed that the both LPF did not exert a significant growth inhibitory characteristics (Fig. 1d). The biofilm inhibitory activity of V-shaped LPF was further confirmed by CLSM and SEM images of pathogenic *C. albicans* biofilms. The V-shaped LPF-exposed *C. albicans* cells showed a reduction in the biofilm confluency on polystyrene surfaces and the inhibitory activity was significantly better compared to non-V-shaped LPF (Figs 1b and e) and nylon membranes (Fig. 1f). The SEM images revealed the majority of the cells in the V-shaped LPF-treated groups were yeast cells, while almost all the cells in the control group were hyphal filaments. Observation suggested that V-shaped LPF most likely prevented an activation of the yeast-hyphae transition switch. We next investigated the impact of V-shaped LPF on hyphal formations in either solid or liquid media. As anticipated, V-shaped LPF compromised hyphal growth in the tested conditions (Fig. 1f).

**Fig. 2:**
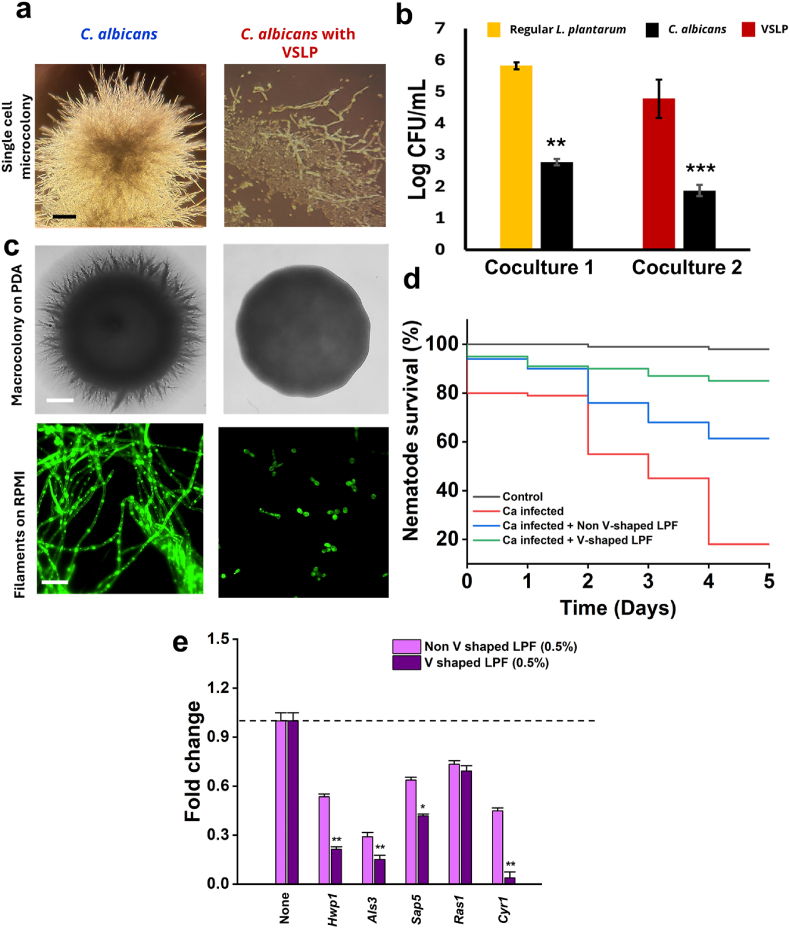
Effect of VSLP and V shaped LPF on microcolony, hyphae, microcolony, biofilm formation and gene expression in *Candida albicans*. (a) Single-cell microcolony formation of *C. albicans* in the presence or absence of VSLP, visualized by light microscope on Ibidi μ-slides after 24 h of coculture in RPMI medium at 37 °C. Scale bar: 50 μm. (b) Colony-forming unit (CFU) enumeration of *C. albicans* cocultured with either VSLP or regular *L. plantarum*. Cocultures were grown in RPMI medium at 37 °C for 24 h, serially diluted, and plated on potato dextrose agar for quantitation. Data represent mean ± SEM from three independent experiments. ∗∗p < 0.01, ∗∗∗p < 0.001 compared with untreated controls. (c) Inhibition of *C. albicans* hyphal and macrocolony phenotypes by VSLP. Upper panel: macrocolony growth on PDA after 48 h incubation. Lower panel: filamentation in RPMI medium visualized by fluorescence microscopy (stained with calcofluor white). Scale bars: 100 μm. (d) Kaplan–Meier survival curves of *Caenorhabditis elegans* infected with *C. albicans* in the presence or absence of *L. plantarum*. Synchronized adult worms were infected with *C. albicans* (ATCC 90028) and treated with either regular *L. plantarum* or VSLP. Survival was monitored daily over 5 days. (e) Gene expression levels of *hwp1, als3, sap5, ras1,* and *cyr1* were analyzed by qPCR after 6 h treatment with 0.5 % V-shaped LPF or non–V-shaped LPF. *act1* was used as the housekeeping gene, and relative fold change was calculated using the CT method. Data are presented as mean ± SD of three independent experiments. Statistical significance compared with untreated control: ∗p < 0.05; ∗*p < 0.01*.

We next compared the antagonistic effect of postbiotic substances (PS) derived from the VSLP vs. unstructured cells. Numerous studies have shown that PS derived from Lactobacilli (grown at regular conditions) show potent antimicrobial properties [[28], [29], [30]], However, the effect of PS generated by the VSLP cells, namely V-shaped LPF, has not been tested yet. Focusing our investigation further on hyphal growth by *C. albicans*, we set up a co-culture assay using the VSLP cells generated onto the ibidi slides (μ-Slide I Luer). While the *C. albicans* cells formed a well-developed microcolony in the control group, they failed to do so following exposure to the VSLP cells (Fig. 2a). This result indicates that *L. plantarum* could suppress the pathogenic microcolony formation. Consequently, the microscopic observation demonstrated the hyphal inhibitory activity of the VSLP cells (Fig. 2a). We also conducted a coculture assay using the liquid RPMI medium by inoculating the VSLP or regular *L. plantarum* cells together with *C. albicans.* In both cases, we found a reduction in *C. albicans* growth, but the reduction was highly significant in the presence of VSLP cells (Fig. 2b). Furthermore, we tested the effect of V-shaped LPF on yeast-to-hyphae transition (Y–H) in *in* *vitro* conditions and hyphal protrusion from macrocolony edges. V-shaped LPF clearly inhibited hyphae in *C. albicans* colony (Fig. 2c) and Y–H transition (Fig. 2c). The findings indicate that the components of V-shaped LPF could serve as a promising anti-hyphal agent. Additionally, we conducted *in-vivo* experiments with *C. elegans*, by infecting the worms with the pathogens, that resulted in the reduction of the nematodes’ lifespan. Our data indicates that supplementation of the VSLP cells prior to an infection of *C. elegans* with pathogenic cells clearly halted pathogenicity resulting in enhanced survival of the infected nematodes. We conducted a time-lapse study and plotted a Kaplan–Meier plot showing the survivability of *C. elegans* (Fig. 2d). qPCR analysis revealed that treatment with V-shaped LPF significantly downregulated the expression of key *C. albicans* virulence-associated genes compared to untreated controls. Specifically, *hwp1* and *als3* were markedly reduced (p < 0.01), while *sap5* showed moderate but significant suppression (*p < 0.05*). Expression of *ras1* was slightly reduced but not statistically significant. Importantly, *cry1*, a central regulator of cAMP signaling, was strongly suppressed (*p* < 0.01), indicating that V-shaped LPF may interfere with pathways critical for biofilm formation and pathogenicity (Fig. 2e).

To further identify the postbiotics produced during V-shaped structuring, we conducted LC-MS analysis and identified almost 55 new metabolites (29 by positive ionization mode and 26 by negative ionization mode) in the LPF extracted from the VSLP cells (V shaped LPF) by LCMS profiling (Table 1). Several of them are considered as potent antibacterials while others like taurolithocholic acid, and spermidine are established biofilm inhibitors of pathogens *Pseudomonas aeruginosa* and *E. coli* K-12 respectively [31,32].

**Table 1:** Bioactive metabolites in V-shaped LPF extracted from *L. planarum* grown at pH 3.5

Name	Molecular Formula	Mass (g/mol)	RT (s)	*m*/*z*
Spermine	C10H26N4	202.2128	1.001	203.2201
Neuraminic acid	C9H17NO8	267.0934	1.418	268.1006
Buthionine sulfoximine	C8H18N2O3S	222.1032	2.085	245.0922
Ketotifen	C_19_H_19_NOS	309.1179	2.169	310.125
Ethyl 2-aminobenzoate	C9H11NO2	165.0789	3.538	188.0681
Desglucocoroloside	C29H44O7	504.3085	4.068	543.2718
2-Octaprenyl-3-methyl-6methoxy-1,4-benzoquinol	C_38_H_60_O_3_	564.4492	4.113	321.1885
Hypoglycin B	C_12_H_18_N_2_O_5_	270.1215	4.15	293.1107
15beta-Hydroxy-7alphamercapto-pregn-4-ene-3,20dione 7-acetate	C_23_H_32_O_4_S	404.2018	4.475	405.2091
Imazamethabenz (para)	C15H18 N2 O3	274.1335	4.481	297.1231
Mycinamicin V	C37H61NO12	711.4253	4.679	378.7019
Hydroxycotinine	C10H12N2O2	192.0903	4.848	215.0796
Tsugaric acid C	C32H50O5	514.3664	4.957	553.3298
Lappaconitine	C32H44N2O8	584.3102	4.963	585.3188
Bonafousine	C35H40N4O3	564.3119	4.972	603.2746
PG(18:1(9Z)/22:5(7Z,10Z,13Z ,16Z,19Z))	C46H79O10P	822.549	5.076	434.2641
PE(20:3(5Z,8Z,11Z)/14:1(9Z) )	C39H70NO8P	711.4882	5.128	394.7076
4beta-Hydroxywithanolide E	C28H38O8	502.2587	5.358	503.2661
Batrachotoxin	C31H42N2O6	538.3059	5.419	539.3131
Buthionine sulfoximine	C8H18N2O3S	222.1033	5.555	245.0925
all-trans-Nonaprenyl diphosphate	C45H76O7P2	790.5087	5.625	434.2174
MG(22:6(4Z,7Z,10Z,13Z,16Z, 19Z)/0:0/0:0)	C25H38O4	402.2826	5.625	425.2719
(17alpha,23S)-17,23-Epoxy29-hydroxy-27-norlanosta-1,8diene-3,15,24-trione	C_29_H_40_O_5_	468.292	5.687	491.2817
Ochrolifuanine A	C29H34N4	438.2805	5.872	439.288
Hericenone H	C_37_H_54_O_6_	594.391	6.022	633.3551
Taurolithocholic acid 3-sulfate	C26H45NO8S2	563.2594	6.185	586.2486
Miotine	C12H18 N2 O2	222.1364	6.683	245.1257
3-Oxo-12,18-ursadien-28-oic acid	C30H44O3	452.3318	6.833	453.3392
3,6-Epoxy-5,5′,6,6′-tetrahydrob,b-carotene-3′,5,5′,6′-tetrol	C_40_H_58_O_5_	618.428	7.429	657.3914
Phenylmercuric Acetate	C_8_H_8_HgO_2_	332.0214	2.266	391.0352
Cortolone-3-glucuronide	C_27_H_42_O_11_	542.2721	4.749	541.2654
Ranolazine	C_24_H_33_N_3_O_4_	427.2459	4.88	472.2446
Cholic acid glucuronide	C_30_H_48_O_11_	584.319	5.548	583.3127
Oxidized dinoflagellate luciferin Oxidized dinoflagellate luciferin 2-	C33H38 N4 O7 C33H36N4O7	602.276	5.643	601.2694
Lithocholate 3-*O*-glucuronide	C_30_H_48_O_9_	552.3291	5.66	551.3225
S-(PGA1)-glutathione	C_30_H_49_N_3_O_10_S	643.3211	5.895	688.3193
Lithocholic acid sulfate	C_24_H_40_O_6_S	456.261	6.092	501.2593
Alvimopan	C_25_H_32_N_2_O_4_	424.2344	6.158	483.2483
Licoricesaponin C2	C_42_H_62_O_15_	806.4113	6.182	865.4252
Androcymbine	C_21_H_25_NO_5_	371.1719	6.28	370.1648
Cyclolinopeptide H	C_56_H_75_N_9_O_9_S_2_	1081.508	6.527	1140.5219
(S)-Nerolidol 3-*O*-[a-LRhamnopyranosyl-(1->4)-a-Lrhamnopyranosyl-(1->2)-[4-(4hydroxy-3-methoxycinnamoyl)-(E)-a-l-rhamnopyranosyl-(1->6)]-b-d-glucopyranoside]	C49H74 O21	998.4792	6.656	997.4729
Prupaside	C_27_H_36_O_12_	552.2253	6.804	551.2176
Bluensomycin	C_21_H_39_N_5_O_14_	585.2501	6.891	584.2431
Vignatic acid A	C_30_H_39_N_3_O_7_	553.2782	6.922	552.2712
Madlongiside C	C_35_H_56_O_10_	636.3872	7.139	681.386
Cyphenothrin	C_24_H_25_NO_3_	375.1821	7.754	374.175
Majoroside F6	C_48_H_82_O_19_	962.5454	7.755	1007.5456
Oxidized Oplophorus luciferin	C25H21 N3 O3	411.1561	7.835	410.1509
Spirasine I	C_22_H_29_NO_3_	355.2129	7.998	354.2057
Orysastrobin	C_18_H_25_N_5_O_5_	391.1879	8.066	390.1826
Nummularine B	C_32_H_41_N_5_O_6_	591.3099	8.303	636.3083
(3b,21b)-12-Oleanene-3,21,28triol 28-[arabinosyl-(1->3)arabinosyl-(1->3)-arabinoside]	C_45_H_74_O_15_	854.4947	8.441	853.4881
Ganglioside GD2 (d18:0/26:0)	C_87_H_157_N_3_O_34_	1788.0481	9.4	939.0246
Prorocentrolide B	C56H85 N O17 S	1075.5541	9.903	1120.5536

**Fig. 3:**
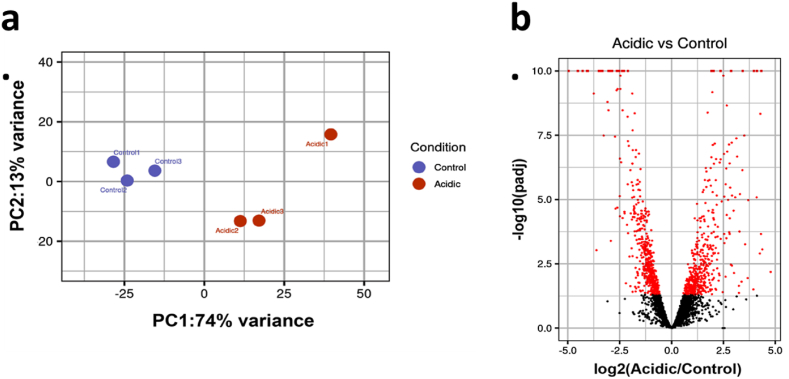
Transcriptomic profiling of V-shaped multicellular structures. (a) Principal Component Analysis (PCA) of *L. plantarum* control (samples in pH 6.5) and acidic (samples in acidic pH 3.5) (b) Volcano plot representing differentially expressed genes in the control and acidic samples. Red dots, padj<0.05, black dots, padj ≥ 0.05. Represent significant genes while the black dots represent the non-significant. (For interpretation of the references to color in this figure legend, the reader is referred to the Web version of this article.)

We next initiated a global transcriptomic analysis to elucidate a mode of antagonistic activity of V-shaped *L. plantarum* cells. The analysis reveals stress-induced gene expression profile in V-Shaped structured cells (VMS). The principal component analysis (PCA) plot provided a clear visualization of gene expression variation between the control and stress adapted cells, with PC1 accounting for 74 % of the variance and PC2 for 13 % (Fig. 3a). The Volcano plot further highlighted significant differences in gene expression in VMS cells, identifying genes with a padj<0.05, many of which with a fold change (FC) > 2.0 (Fig. 3b). A general comparison of the sequenced libraries from *L. plantarum* VMS cells and control cells cultured at pH 6.5 revealed a total of 994 differentially expressed genes (DEGs) of which 462 were upregulated and 532 genes were downregulated (Supplementary data 1).

**Fig. 4:**
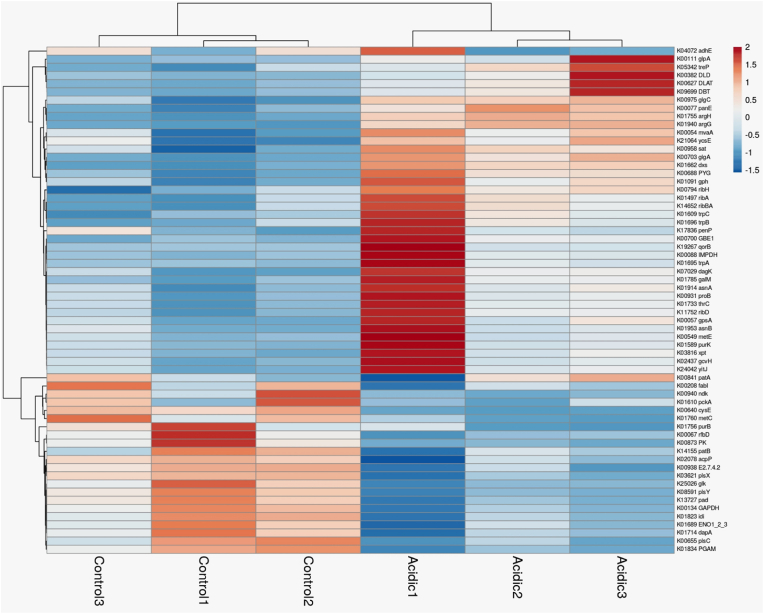
Heat map representing the differentially regulated genes involved in the biosynthesis of secondary metabolites.

From GSEA performed on all genes, we found that the most upregulated genes were associated with the biosynthesis of metabolic pathways. Of specific interest, 63 genes of the total gene set, involved in the biosynthesis of secondary metabolites, were found to be significantly regulated (Fig. 4 and supplementary data 1). Additionally, genes involved in amino acid metabolism (e.g., *brnQ, argH, argG, secA*), oxidation-reduction processes (e.g., *qorB, panE2, pox5_4*), carbon metabolism (e.g., *manP, sorD, bglH*), and aromatic amino acid metabolism (e.g., *trpB, trpA*) showed significant upregulation. Conversely, downregulated genes included those encoding ribosomal proteins and translational regulatory activity (e.g., *rpsF, rpsR, rpsP, rplS, rplG, rpsT*). To further investigate the potential secondary metabolite-producing genes in *L. plantarum*, we conducted an antiSMASH analysis on its reference genome (*L. plantarum*, GenBank: CP028221.1). AntiSMASH analysis identifies biosynthetic gene clusters (BGCs), which typically contain all the genes necessary for the production of specialized or secondary metabolites [33]. Our analysis of the reference genome revealed four protocluster regions of BGCs encoding genes for metabolites in the classes of terpenes, cyclic-lactone autoinducers, ribosomally synthesized and post-translationally modified peptide products (RiPP-like), and type III polyketide synthase (T3PKS). Each BGC contained genes encoding core biosynthetic components, additional biosynthetic enzymes, transport proteins, regulatory elements, and other unspecified functions (data not shown).

From overall transcriptomic data, we identified four core biosynthetic genes—*crtN, agrB, lagD,* and *mvaS*—associated with their respective BGCs. Among these, *lagD* (RiPP-like) showed no significant change in expression, with a fold change (FC) of 0.526, while *agrB* (cyclic-lactone autoinducer) was significantly downregulated, with an FC of −2.274. Additionally, among the core biosynthetic genes identified, *pox5_3* (cyclic-lactone autoinducer) showed significant upregulation with an FC of 2.541, whereas the other genes exhibited no significant changes in expression levels (Table 2).

**Table 2:** The core biosynthetic gene clusters (BGCs) and their fold change extracted from the reference genome *L. plantarum* (GenBank: CP028221.1).

Biosynthetic gene clusters	Fold change	padj
**Terpene**		
**Core genes**		
***crtN***	**−0.040**	**0.950**
**Other genes**		
***crtM***	**0.724**	**1.491E-01**
***menH2***	**0.723587**	**0.313333293**
***purB***	**−0.67885**	**0.027614697**
***pdhD2***	**−0.96137**	**0.043569085**
**Cyclic-Lactone-Autoinducer**		
**Core genes**		
***agrB***	**−2.274**	**2.98E-05**
**Other genes**		
***andAa***	**−0.853**	**0.002507**
***pox5_3***	**2.541**	**7.66E-06**
**Ripp-like**		
**Core genes**		
***lagD***	**0.526**	**0.251585**
**Other genes**		
***agrA_4***	**−1.35281**	**4.13E-05**
***lcnD***	**0.930**	**0.08508**
**T3PKS**		
***mvaS***	**−0.090**	**0.800808**
**Other genes**		
***rasP***	**0.468**	**0.286242**
***yjjG***	**−1.421**	**0.00045**
**ACJMCBPC_00689**	**0.142**	**0.742042**
***plsC***	**−0.751**	**0.031102**

**Fig. 5:**
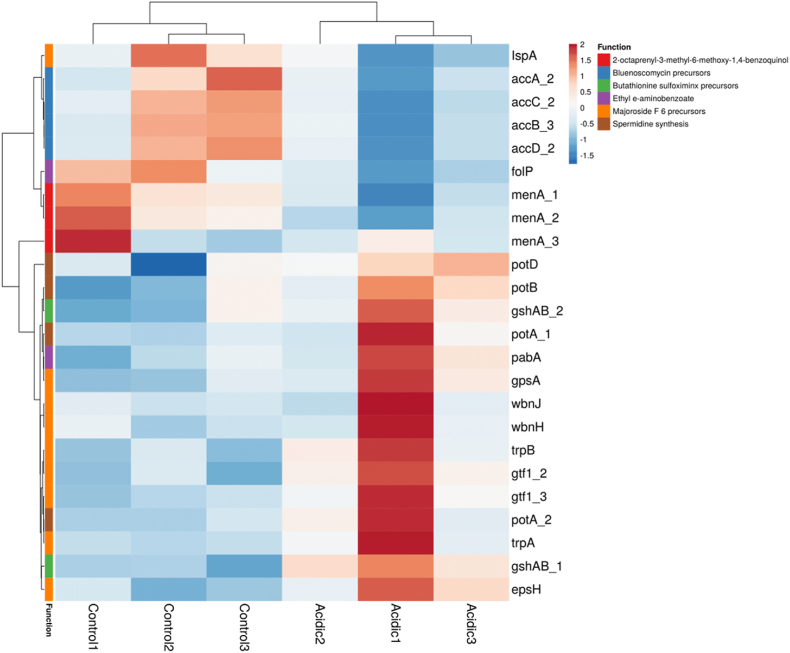
Heat map of precursor genes involved in the biosynthesis of secondary metabolite in *L. plantarum*.

Additionally, we identified a set of genes that serve as precursors for secondary metabolite synthesis (Table 3). Among these, genes involved in the synthesis of the polyamine spermidine exhibited significant upregulation (Fig. 5). Specifically, *potA_2* (FC: 3.597), encoding spermidine ATP-binding protein, and *potD* (FC: 3.59), encoding spermidine-binding periplasmic protein, were highly expressed. These genes play a crucial role in bacterial growth, biofilm formation, and the production of important bacteriocins [34]. They also exhibit potential antagonistic activity against *Escherichia coli* K-12 [31]. Other key precursor genes that showed differential expression in our transcriptomic data included *trpA* (FC: 1.697), responsible for synthesizing the tryptophan alpha chain; *gpsA* (FC: 1.684), encoding glycerol-3-phosphate dehydrogenase; and a hypothetical gene (*ACJMCBPC_01286*, FC: 1.458), which encodes farnesyl diphosphate synthase (https://www.kegg.jp/entry/lpl01110). These genes are associated with the lipid biosynthesis pathway and have evolutionary significance in the synthesis, secretion, and transport of secondary terpenoid-class metabolites. This pathway may ultimately contribute to the secretion of majoroside F6 (https://www.kegg.jp/entry/lpl01110*).*

**Table 3:** Fold change of precursor genes involved in the biosynthesis of secondary metabolites in the V-shaped structured *L. plantarumcells.*

Genes	Function	Fold change	padj
Spermidine synthesis			
*potA_2*	Spermidine/putrescine_import_ATP-binding_protein_PotA	3.5974	0.07660
*potD*	Spermidine/putrescine-binding_periplasmic_protein	3.597	0.19964
*potB*	Spermidine/putrescine_transport_system_permease_protein_PotB	0.359	0.02164
*potA_1*	Spermidine/putrescine_import_ATP-binding_protein_PotA	1.296	0.01324
Butathionine sulfoximinx precursors			
*gshAB_1*	Glutathione_biosynthesis_bifunctional_protein	1.125	0.06009
*gshAB_2*	Glutathione_biosynthesis_bifunctional_protein	0.575	0.08482
Ethyl e-aminobenzoate			
*pabA*	Aminodeoxychorismate_synthase_component_2	0.808	0.23291
*folP*	Dihydropteroate_synthase	1.312	0.01290
2-octaprenyl-3-methyl-6-methoxy-1,4-benzoquinol			
*menA_1*	1 %2C4-dihydroxy-2-naphthoate_octaprenyltransferase	−0.969	0.0150
*menA_2*	1 %2C4-dihydroxy-2-naphthoate_octaprenyltransferase	−1.032	0.00173
*menA_3*	1 %2C4-dihydroxy-2-naphthoate_octaprenyltransferase	−1.351	0.78200
Bluenoscomycin precursors			
*accB_3*	Biotin_carboxyl_carrier_protein_of_acetyl-CoA_carboxylase	−0.123	0.25913
*accC_2*	Biotin_carboxylase	−1.426	0.21863
*accD_2*	Acetyl-coenzyme_A_carboxylase_carboxyl_transferase_subunit_beta	−1.500	0.05313
*accA_2*	Acetyl-coenzyme_A_carboxylase_carboxyl_transferase_subunit_alpha	−1.444	0.05858
Majoroside F 6 precursors			
*trpB*	Tryptophan_synthase_beta_chain	−1.359	0.00450
*trpA*	Tryptophan_synthase_alpha_chain	1.617	0.00505
*gpsA*	Glycerol-3-phosphate_dehydrogenase_[NAD(P)+]	1.684	0.00712
ACJMCBPC_01286	Farnesyl_diphosphate_synthase	1.458	0.59972
*wbnJ*	O-antigen_biosynthesis_glycosyltransferase_WbnJ	−0.160	0.25079
*wbnH*	O-antigen_biosynthesis_glycosyltransferase_WbnH	0.941	0.16185
*epsH*	Putative_glycosyltransferase_EpsH	1.074	0.02709
*gtf1_2*	Glycosyltransferase_Gtf1	0.772	0.03801
*gtf1_3*	Glycosyltransferase_Gtf1	0.704	0.00035

**Fig. 6:**
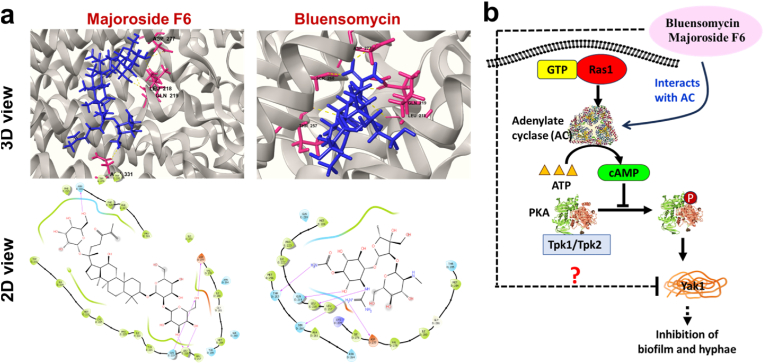
Predicted interaction of bioactive compounds with adenylate cyclase and their proposed mode of action in *Candida albicans*. (a) 3D and 2D docking interactions of Majoroside F6 and Bluensomycin with adenylate cyclase, showing key amino acid residues involved in binding. (b) Proposed mechanism of action: Majoroside F6 and Bluensomycin interact with adenylate cyclase, leading to reduced cAMP production in the Ras1–cAMP–PKA pathway, thereby inhibiting biofilm formation and hyphal growth.

Finally, to substantiate the hyphal growth or biofilm inhibitory activities we observed in Fig. 1, we conducted a computational docking of all these 55 ligands with the enzyme, adenylate cyclase (AC). Adenylate cyclase is a dynamic modulator of the Ras-adenylate cyclase pathway that efficiently regulates *C. albicans* filamentation features. Also, it appears that the Ras-adenylate cyclase pathway controls the hyphal switch in fungal pathogens [[35], [36]]. Docking results identified 2 compounds, namely bluensomycin and majoroside that showed remarkable binding efficiencies with adenylate cyclase (Fig. 5) with a Glide docking score of −10.265 and −10.89 Kcal/Mol, respectively; such docking score appears as significantly better than the standard ligand 2′,5′-Dideoxyadenosine 3′-triphosphate tetrasodium salt (−4.86 kcal/mol). The findings also support the transcriptomic data with an upregulation of genes involved in the biosynthesis of bacteriocins. Based on those findings, we suggest that the bluensomycin and majoroside F6 function as competitive inhibitors which may achieve hyphal or biofilm inhibition through Ras-adenylate cyclase pathway by inactivating AC (Fig. 6a and b).

## 4 Conclusion

The work characterizes the antagonistic capacity of postbiotic substances derived from the functionalized probiotic cells (such as VSLP cells) against pathogenic yeast. Overall, our analyses provide valuable insights into the genetic basis of secondary metabolite production. The identification of several biosynthetic gene clusters (BGCs) and key precursor genes highlights the potential for the production of diverse metabolites, such as terpenes and cyclic-lactone autoinducers. The upregulation of genes involved in spermidine synthesis suggests their role in bacterial growth, biofilm formation, and bacteriocin production, while genes associated with lipid biosynthesis may contribute to the synthesis of important secondary metabolites like majoroside F6. Furthermore, the investigation pinpoints several metabolites as potent Ras-adenylate cyclase pathway inhibitors in *C. albicans*. Further study is warranted to explore the broad-spectrum antimicrobial or additional biological activities of the metabolites identified in this study.

## CRediT authorship contribution statement

**Satish Kumar Rajasekharan:** Writing – review & editing, Writing – original draft, Validation, Supervision, Project administration, Methodology, Investigation, Conceptualization. **Athira Venugopal:** Writing – review & editing, Methodology, Investigation. **Hamitha Chinganadi Hameed:** Investigation, Methodology, Writing – review & editing. **Jennessa Jacob:** Writing – review & editing, Visualization, Formal analysis, Data curation. **Vinothkannan Ravichandran:** Methodology. **Doron Steinberg:** Writing – review & editing, Formal analysis, Data curation. **Adi Faigenboim:** Validation, Methodology. **Chaitany Jayprakash Raorane:** Methodology, Investigation. **Moshe Shemesh:** Writing – review & editing, Validation, Project administration, Funding acquisition, Data curation.

## Declaration of competing interest

The authors declare that they have no known competing financial interests or personal relationships that could have appeared to influence the work reported in this paper.

## Data Availability

Data will be made available on request.
